# Transcriptional, biochemical, and histochemical response of resistant and susceptible cultivars of *Brassica juncea* against *Albugo candida* infection

**DOI:** 10.3389/fpls.2024.1426302

**Published:** 2024-08-01

**Authors:** Prajjwal Rai, Lakshman Prasad, Samridhi Mehta, Prashant Yadav, Anubhuti Sharma, Deep Narayan Mishra

**Affiliations:** ^1^ Division of Plant Pathology, ICAR-Indian Agricultural Research Institute, New Delhi, India; ^2^ ICAR-National Institute for Plant Biotechnology, New Delhi, India; ^3^ ICAR-Directorate of Rapeseed-Mustard Research, Bharatpur, India

**Keywords:** *Albugo candida*, biotrophic, *Brassica juncea*, PR protein genes, biochemical, callose, Reactive Oxygen Species

## Abstract

White rust disease caused by a biotrophic oomycete *Albugo candida* is one of the most serious impediments in realizing the production potential of *Brassica juncea*. Due to the obligate nature of the pathogen, R-gene-based resistance is unstable as the newer virulent races emerge quickly. For this, a deep understanding of the molecular basis of resistance is essential for developing durable resistant varieties. In this study, we selected one susceptible cultivar, ‘Pusa Jaikisan’ and its single *R* gene based resistant NIL, ‘Pusa Jaikisan *WRR* as the source of understanding the defense mechanism in *B. juncea* against *A. candida*. Comparative histochemical analysis at 12 dpi showed higher callose deposition in the resistant cultivar than in the susceptible which hints towards its possible role in defense mechanism. Based on the biochemical markers observation, total protein was found to have a negative correlation with the resistance. The antioxidant enzymes (POX, CAT, and SOD) and non-enzymatic ROS scavenging compounds such as polyphenols and proline showed a positive correlation with the white rust resistance. Polyphenol Oxidase (PPO) total chlorophyll and total carotenoids were also found to be more abundant in the ‘Pusa Jaikisan WRR’. Based on the heat map analysis, PAL was identified to be the comparatively most induced enzyme involved in the defense mechanism. The polyphenol oxidase, total chlorophyll and total carotenoids were also found to show higher activity in the ‘Pusa Jaikisan *WRR*’. Furthermore, to study the defense response of ‘Pusa Jaikisan *WRR*’ compared to ‘Pusa Jaikisan’ against *A. candida* infection, the gene expression analyses of salicylic acid (SA)-marker PR protein genes (*PR1* and *PR2*) and jasmonic acid (JA)-marker PR protein genes (*PR3* and *PR12*) were done by qRT-PCR. Based on the results, *PR2* emerged as the best possible gene for defense against *A. candida* followed by *PR1*. *PR3* and *PR12* also showed positive correlation with the disease resistance which may be due to the JA pathway acting complementary to the SA pathway in case of *B. juncea*-*A. candida* interaction. This provides evidence for the JA-SA hormonal crosstalk to be synergistic in case of the white rust resistance.

## 1 Introduction

Regarding economic significance, oilseeds are second only to cereal crops. The global trends of area, production, and yield in the year 2021-22 were 41.95 mha, 88.35 mt and 2110 kg/ha, respectively (ICAR-DRMR, 2023). In India, rapeseed mustard has a share of one-fourth of total edible oil production and cultivated area. Out of this, Indian mustard [*Brassica juncea* (L.) Czern. And Coss.] has the lion’s share (about 36%) in India’s edible oil kitty (ICAR-DRMR, 2023). India imported 1.3 mt of edible oil in June 2023, which is alarming (The Solvent Extractors’ Association of India, 2023). On the other hand, many biotic and abiotic stresses are serious impediments to realizing the potential production of *B. juncea.* Among all these, white rust disease caused by *Albugo candida* is a globally important problem. It is known to cause losses ranging from 1% to 90% which depends on a multitude of factors such as plant population, host genotype, nutrition (Saharan and Verma, 1992). Chemicals such as Mancozeb and a combination of metalaxyl and mancozeb have proven to be quick and effective methods of disease management but they come with their own set of economically and environmentally negative spillovers (Gairola and Tewari, 2019). Breeding for disease resistance is the best strategy for managing the disease (Ren et al., 2016). *A. candida* being an obligate biotroph shares a strong co-evolutionary relationship with the host and this enables quick racial evolution and breaks the already existing *R* gene-based resistance. Given these factors, genetic engineering for the broad-spectrum non-host resistance (NHR) genes such as pathogenesis-related protein (PRs) genes is the most apt method for managing the dreaded white rust (Grover, 2012; Rai et al., 2023). Their expression pattern can be useful in identifying their role in resistance and future use. Further, a set of biochemical markers such as antioxidant enzymes, act as surrogate measures of resistance. These can be coupled with the histochemical parameters such as callose deposition in the inner walls which acts as determinants of resistance activation (Kaur et al., 1984). Together these three can be utilized for developing a thorough understanding of the defense mechanism against *A. candida* and developing durable transgenics.

As plants are sessile, they have evolved an array of mechanisms to thwart the growth of pathogens upon invasion. Among these, callose deposition, induction of pathogenesis related proteins (*PR*), catalase, superoxide dismutase and peroxidase, production of phenolics, and reactive oxygen species (ROS) are the foremost (Torres et al., 2006; Doughari, 2015). When a plant encounters the pathogen, it initiates a two-fold resistance response. The primary or basal response is mediated by recognizing the pathogen-associated molecular patterns (PAMPs) through the PAMP recognition receptors (PRRs). This is termed as the PAMP-triggered immunity (PTI) (Jones and Dangl, 2006). PTI induces the production of *PR* proteins, ROS, cell wall reinforcement and antimicrobial compounds produced through the mitogen-activated protein kinases (MAPKs) mediated signaling pathways (Bigeard et al., 2015). Cell death caused by ROS is usually favored by the pathogen in plant-necrotroph interaction but is an important component of disease resistance in the case of plant-biotroph interaction (Govrin and Levine, 2000; Pietrowska et al., 2015. Cell death is a necessary process, but too much can be detrimental to the plant itself. Hydrogen peroxide (H_2_O_2_) is one of the most important components of ROS and accumulates after the plant recognizes the pathogen. Hydrogen peroxide is converted into water and oxygen in peroxisome by catalase (CAT) (EC 1.11.1.6) (Afiyanti and Chen, 2014), whereas peroxidases (POX) (EC 1.11.1.7) reduce H_2_O_2_ along with many phenolics and non-phenolics substrates. Superoxide dismutase (SOD) (EC 1.15.1.1) negates the harmful effect of superoxide radicals by dismutation into oxygen and water (Bittner et al., 2017). The polyphenols play an important role by neutralizing the ROS, while polyphenol oxidase (PPO) (EC 1.10.3.2) catalyzes the oxygen-dependent oxidation of polyphenols to quinones. Together these two maintain ROS-homeostasis, which helps in plant defense (Arora et al., 2000; Kaur et al., 2017). Proline is an important non-enzymatic antioxidant that can also neutralize ROS and act as a balancing wheel (Verbruggen and Hermans, 2008). Phenylalanine ammonia-lyase (PAL) (EC 4.3.1.24) is one of the most crucial enzymes in the defense pathway. It catalyzes the deamination process of phenylalanine into secondary phenylpropanoid metabolism (Hahlbrock and Scheel, 1989). For producing these enzymes, the protein is broken down hence decreasing its concentration upon resistance activation (Bolwell and Wojtaszek, 1997). The relationship of chlorophyll and carotenoids with resistance have not been strongly ascertained and need future studies.

*PR* proteins along with hormonal responses induced by complex signaling pathways are the major determinants of plant resistance and are strongly induced upon pathogen inoculation (van Loon et al., 2006; Grover, 2012). These are small proteins which are accumulated and induced in response to an array of biotic and abiotic stresses (Ebrahim et al., 2011). At present, *PR* proteins are classified into 17 families and among them, the major ones are *PR1* (antifungal), *PR2* (β-1,3-glucanase), *PR3* (chitinase), *PR5* (thaumatin-like protein), *PR9* (peroxidases), *PR12* (plant defensin), *PR13* (thionins) (van Loon et al., 2006). Regarding hormonal signaling-mediated resistance, salicylic acid (SA) and jasmonic acid/ethylene (JA/ET) are important components. They are thought to be mutually antagonistic. The JA/ET pathway is initiated upon attack by a necrotroph while in the case of a biotroph, the SA pathway is predominant. Multiple reports hint at a positive role of JA/ET in the case of biotroph attacks where they complement the SA pathway (Glazebrook, 2005). *PR1*, *PR2* and *PR5* are the SA-marker genes, while *PR3*, *PR12* and *PR13* are the JA/ET-marker genes (Thomma et al., 2001). As, *B. juncea* suffers from a heterogeneous group of pathogens which includes both biotrophs and necrotrophs, the signaling pathway genes may help in developing broad-spectrum durable resistance. Several transgenic plants over-expressing *PR* genes have been developed against *B. juncea* pathogens imparting disease resistance (Huang et al., 2016; Dong et al., 2017; Ali et al., 2018).

A comparative study of the disease resistance factors in resistant and susceptible cultivars of *B. juncea* is essential to develop durable transgenics. Here we have chosen *B. juncea* cv. Pusa Jaikisan (susceptible) and *B. juncea* cv. Pusa Jaikisan *WRR* (resistant) (Arora et al., 2019) source for white rust resistance and studied the disease progression, callose deposition after infection, defense-related enzymes and non-enzymatic compounds, and the role of *PR* genes as markers of the hormonal signaling pathway in resistance against *A. candida*.

## 2 Materials and methods

### 2.1 Plant materials and fungal materials

Two cultivars of Indian mustard (*B. juncea*), ‘Pusa Jaikisan’ and ‘Pusa Jaikisan *WRR*’ were used as plant materials and the seeds were obtained from the ICAR-Directorate of Rapeseed Mustard Research, Bharatpur (India). These experiments were carried out from November 2022 to March 2023 at the Division of Plant Pathology, ICAR- Indian Agricultural Research Institute, New Delhi (India). The research complied with the institutional, national, and international guidelines and legislation.

*Albugo candida* of New Delhi isolate *Ac-Dli* was originally isolated from ‘Pusa Jaikisan’ (*B. juncea*) from the field of Division of Genetics, ICAR- Indian Agricultural Research Institute. For maintaining the *Ac-Dli* isolate, seedlings of ‘Pusa Jaikisan’ were used. Seven-day-old plants were inoculated by spraying *Ac-Dli* with the concentration of 1 X 10^5^ zoosporangia/ml, followed by incubating in a moist chamber for 24 h at 22^0^C under the dark conditions and then in a growth chamber for 16 h light and 8 h dark at 21^0^C, with regular irrigation. The process was repeated sufficiently for maintenance. The isolate *Ac-Dli* was maintained in the Division of Plant Pathology, ICAR- Indian Agricultural Research Institute.

### 2.2 Inoculation test

Seeds of ‘Pusa Jaikisan’ and ‘Pusa Jaikisan *WRR*’ were sown in trays in three replications each and were kept under 16 h light alternated with 8 h dark at 21^0^C. Seven-day-old plants were inoculated by spraying *Ac-Dli* with a concentration of 1 X 10^5^ zoosporangia/ml. To confirm the successful inoculation, plants were incubated in a dark growth chamber for 24 h at 22^0^C under dark conditions and then in a growth chamber for 16 h light and 8 h dark at 21^0^C, with regular irrigation. After 12 days, ‘Pusa Jaikisan’ showed characteristic white-colored zoosporangial pustules on both the adaxial and abaxial surface of the cotyledons, while no symptoms were seen on the ‘Pusa Jaikisan *WRR*’ on either side.

The susceptibility of ‘Pusa Jaikisan’ and resistance of ‘Pusa Jaikisan *WRR*’ were confirmed by maintaining the plants in a dark growth chamber for 24 h at 22^0^C with 100% humidity. The plants were then moved into a growth chamber and provided controlled conditions for 16 h light and 8 h dark at 21^0^C, with regular irrigation. For gene expression profiling and biochemical assays, 14-day-old seedlings were inoculated by spraying *Ac-Dli* with a concentration of 1 X 10^5^ zoosporangia/ml. The infected leaf samples were harvested from ‘Pusa Jaikisan’ and ‘Pusa Jaikisan *WRR*’ at 12, 24, 48, 72 and 96 hpi, snap chilled in liquid nitrogen and stored at -80^0^C for further analyses.

### 2.3 Disease parameters

Disease resistance or susceptibility were assayed on the leaves of the three plants at 12 dpi. A white rust disease scoring scale of 0 to 9 was used to characterize the plants in which 0, 1, 3, 5, 7 and 9 correspond to 0, <5, 5-10, 11-25, 26-50 and >50% diseased area, respectively (Bits et al., 2018). The diseased area comprised white-colored zoosporangial pustules. The experiment was carried out in three replications and for a single replication, five leaves were taken from each plant.

### 2.4 Confocal microscopy

Callose deposition near plasmodesmata was observed according to Zavaliev and Epel (2014). For tissue staining and sample preparation, the leaf sample was collected at 12 dpi from both ‘Pusa Jaikisan’ and ‘Pusa Jaikisan *WRR*’. The entire leaf was cut by holding it along the petiole and submerged in 96% ethanol solution. The container having the sample was covered and sealed with parafilm. Incubation was done at room temperature (RT) to bleach the sample. The bleached leaf was removed from the ethanol solution. The leaf was placed on a flat surface and cut strips 5 mm wide using a razor blade. Utmost care was taken to sample the same regions of both leaves as there may be differences in callose deposition in different regions. The sample was then rehydrated by sinking the strips with the help of DDW with 0.01% Tween-20 followed by incubation at RT for 1h. Strips were removed from DDW and placed in a 25 ml glass tube filled to 1/3 with aniline blue solution by using a wire loop. The vacuum desiccator was used for 10 min to get good dye penetration. Tubes were wrapped with aluminum foil and incubated at RT for 2 h. Specimens were then taken for microscopy and were mounted on microscopic slides and observed under a confocal microscope at ICAR- Indian Agricultural Research Institute. (Model: Leica TCS SP52). Configuration of confocal microscopy was built in a single-track mode. The 405 nm diode laser was used for excitation, while the 475-525 nm band-passed emission filter for the aniline blue fluorescence. A white pseudo color was used for aniline blue emission. The important area under the microscope showing disease progression was captured by a camera mounted on the system and photomicrographs were processed in Image J software (Collins, 2007).

### 2.5 Biochemical assays

The biochemical assay was carried out from the infected sample at 0, 12, 24, 48, 72 and 96 hpi. The enzyme extract was prepared by homogenizing 200 mg of leaf sample in liquid Nitrogen. Ice cold 1.5 ml of 50mM Sodium phosphate buffer (pH 7.0), 2mM EDTA, 5mM β-mercaptoethanol and 4% Polyvinylpyrrolidone (PVP) were added to the homogenized powder (except for TPC where no PVP was used). The homogenate was centrifuged at 3000 rpm for 30 min at 4^0^C. The supernatant was stored in aliquots at -40^0^C. This enzyme extract was used for total protein, polyphenols, PPO, PAL, CAT, SOD, and POX estimation.

#### 2.5.1 Protein content

The total protein content was determined by using the Bradford's approach (1976). The bovine serum albumin served as the standard. The assay combination included 2 ml Bradford reagent, 990 µl water, and 10 µl enzyme extract. The absorbance was recorded at 595 nm by using a UV-visible spectrophotometer. Total protein content was expressed as mg/g dry weight.

#### 2.5.2 POX activity

The POX activity was determined by following the Chance and Maehly (1955) methodology. In this, three solutions; Solution A; Sodium phosphate buffer (100mM, pH 7.0), Solution B: 10 mM H_2_O_2_ solution and Solution C: 20mM guaiacols were freshly prepared. 1.5 ml Solution A and 0.12 ml Solution B were taken in a cuvette. Absorbance increment was recorded at 470 nm wavelength for 3 min at intervals of 1 min each. The reaction mixture without enzyme served as blank. Tetra guaiacol (26.6 mM^-1^cm^-1^) was used as the extinction coefficient of the oxidation product.

#### 2.5.3 CAT activity

CAT activity was determined by following the methodology described by Aebi (1984). 0.1 ml enzyme extract was mixed with 1.5 ml solution A and 0.4 ml distilled water. The decrease in absorbance was recorded for 3 min at 30 s intervals at 240 nm just after the addition of 1ml solution B. The extinction coefficient of H_2_O_2_ (39.4 mM^-1^ cm^-1^) was used for quantification.

#### 2.5.4 Superoxide dismutase activity

Superoxide dismutase activity was measured following the Beauchamp and Fridovich (1971) method. The assay solution contained 27 ml of 50 mM sodium phosphate buffer (pH 7.8), 1 ml of 1.72 mM nitroblue tetrazolium (NBT), 1.5 ml of 201 mM methionine, and 0.75 ml of 1% Triton X-100. To prepare the enzyme extract, plant tissues were homogenized in the sodium phosphate buffer and centrifuged at 10,000 g for 15 minutes at 4°C. The supernatant was used for the assay. For the assay, 1 ml of the solution mix, 0.1 ml of enzyme extract, and 0.03 ml of 20 μM riboflavin were combined in a test tube and incubated in a foil-lined box with a 40W fluorescent bulb for 10 minutes. The reaction was terminated by switching off the light, and absorbance was measured at 560 nm. A reaction mixture without the enzyme extract served as the blank, representing 100% NBT reduction.

#### 2.5.5 Total polyphenol content

The total polyphenol content was determined by Singleton and Rossi (1965) method. A diluted test sample (0.5 ml) was reacted with 0.2 ml Folin-Ciocalteau reagent, and the reaction was neutralized with sodium carbonate (0.5 ml). 10 ml distilled water was used to make up the volume. The absorbance of the blue color was measured at 760 nm. The gallic acid standard curve was used for quantification.

#### 2.5.6 PPO activity

Polyphenol oxidase activity was measured following Meyer et al. (1998) method. Fresh leaves (0.2 g) were homogenized in 2 ml of pH 6.5 0.1 M sodium phosphate buffer and centrifuged at 16,000 rpm for 15 min at 4^0^C. The enzyme was obtained from the supernatant. 200 µl of enzyme extract and 1.5 ml of 0.1 phosphate buffer (pH 6.5) were used for the process. The reaction was started by using 200 µl of 0.01 M catechol, and the enzyme activity was measured at 495 nm/min/mg protein.

#### 2.5.7 PAL activity

PAL activity was measured following the methodology described by Edward and Kessmann (1992). 25 mM tris buffer (pH 8.8) was used as the homogenization buffer. After adding 0.1 ml of enzyme extract and 0.4 ml of 0.05 M tris HCL buffer (PH 8.8) having 0.2 mM phenylalanine, the reaction mixture was incubated in a water bath at 37^0^C for 60 min. To stop the reaction, 0.1 ml of 0.5 N HCL was added. 2 ml of toluene was used to extract the trans-cinnamic acid. The absorbance was measured at 412 nm followed by calculating enzymatic activity.

#### 2.5.8 Proline content

The total proline content was calculated following the methodology described by Koca et al. (2007). 0.2 g of fresh leaf sample was homogenized in 2 ml of 3% w/v distilled water sulfosalicylic acid and centrifuged at 10,000 rpm for 30 min at 4°C. 1 ml of supernatant was combined with 1 ml of glacial acetic acid and 1 ml of acid ninhydrin reagent and incubated for 1 hour at 100°C in a water bath before being immediately plunged into an ice bath to stop the reaction. 2 ml of toluene was after that transferred to each reaction mixture tube. The chromophore was finally extracted by vigorously swirling on a vortex mixer. A spectrophotometer was used to measure the absorbance of the chromophore-comprising toluene layer at 520 nm.

#### 2.5.9 Chlorophyll content

The chlorophyll content ( mg/g fresh weight) was calculated by placing the 10 mg of fresh tissue in a 2 µl microcentrifuge and 700 ul of preheated DMSO was added to the tube containing the leaf tissue. For 30 min, the tube was placed in a 65^0^C water bath. Following incubation, 300 µl of DMSO was added to achieve a volume of 1 ml. The absorbance was measured at three different wavelengths: 470 nm, 645 nm, and 663 nm. Arnon’s equations were used to calculate the chlorophyll content (Richardson et al., 2002). Lichtenthaler and Welburn’s (1983) methodology was used to determine the total carotenoid content (mg/g fresh weight) by using the same enzyme extract.

### 2.6 Gene expression profiling

For the gene expression profiling, RNA was isolated from the infected samples at 0, 12, 24, 48, 72 and 96 hpi by RNeasy^®^ Plant Mini Kit QIAGEN (Germany) as described by the manufacturer’s protocol. For determining the purity and concentration of RNA, the Nanodrop spectrophotometer (NanoDrop2000 Thermo Scientific, Wilmington, DE) was used. First-strand cDNA was synthesized using total RNA isolated from the leaf samples by Promega ImProm-IITM Reverse Transcription system (USA) following the manufacturer’s protocol. The oligoanalyzer software was used for designing the primers of *PR1*, *PR2*, *PR3* and *PR12* genes. For this purpose, the conserved regions were used (**Table 1**). The genes *PR1* and *PR2* are SA-marker genes, while *PR3* and *PR12* are JA/ET-marker genes and are used for ascertaining their role in the defense pathway of *B. juncea*-*A. candida* interaction. The qRT-PCR reaction mixture contained 2 µl of cDNA, 5 µl of SYBR green qRT-PCR master mix (Takara kit, Japan) and 0.5 µl of each primer. Thermal conditions were kept at 94^0^C for 3 min, followed by 40 cycles of 94^0^C for 30 s, 60^0^C for 30 s, and 72^0^C for 30 s. Alpha-tubulin was used as the housekeeping gene which acted as internal control. The reactions were performed in triplicates and repeated using three biological replicates and the delta CT method was used to analyze the relative expression level of each gene (Livak and Schmittgen, 2001). Fold changes with p ≤ 0.05 were taken as the significant level of expression.

**Table 1:** List of primers used for the qRT-PCR analysis of *PR1*, *PR2*, *PR3* and *PR12* genes at 0, 12, 24, 48, 72 and 96 hpi.

S. No.	Primer name	Primer sequence
1.	*PR1*-For	5’-GAACACGTGCAATGGAGAATG-3’
2.	*PR1*-Rev	5’-CCATTGTTACACCTCGCTTTG-3’
3	*PR2*-For	5’-CGTCTCTCTACAATTCGCTCTG-3’
4.	*PR2*-Rev	5’-CGATATTGGCGTCGAATAGGT-3’
5.	*PR3*-For	5’-AAGACCAGGTTCTTGCCTTC-3’
6.	*PR3*-Rev	5’-TCCGGTACACTCCCTACTATTC-3’
7.	*PR12*-For	5’-CAATGGTGAAAGCGCAGAAG-3’
8.	*PR12*-Rev	5’-AGGTGATGCACTGGTTCTT-3’
9.	*Alpha-tubulin*-For	5’-GCCTCGTCTCTCAGGTTATTTC-3’
10.	*Alpha-tubulin*-Rev	5’-TGAAGTGGATTCTTGGGTATGG-3’

These primers are derived from the conserved region of *B. juncea*.

### 2.7 Statistical analyses

A complete randomized design was set up for the experiment and the disease was scored by taking three replicates from each infected plant. The results were analyzed by using the one-way analysis of variance (ANOVA) p ≤ 0.05 level of significance through Microsoft Excel.

## 3 Results

### 3.1 Disease severity

**Figure 1:**
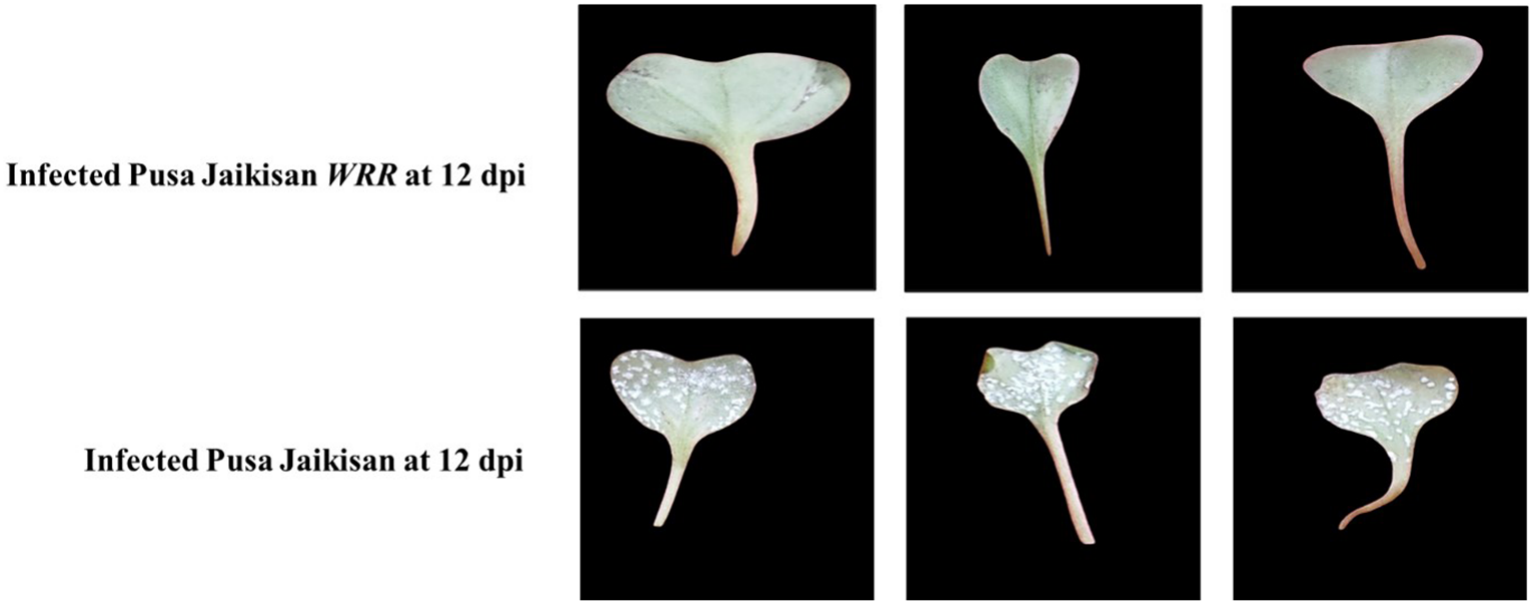
Comparative disease parameters in *B. juncea* cv. Pusa Jaikisan and *B. juncea* cv. Pusa Jaikisan *WRR*. The experiment was carried out in three replications Diseased area was more than 50% in the case of ‘Pusa Jaikisan *WRR*’, while it was 0% for ‘Pusa Jaikisan’.

Disease severity was recorded at 12 dpi. ‘Pusa Jaikisan’ was found to be highly susceptible with more than 50% area covered by pustules and a rating score of 9, while ‘Pusa Jaikisan *WRR*’ had no pustules on both adaxial and abaxial surface with 0% leaf area covered and rating of 0 on the 0-9 scale (**Figure 1**) (Bits et al., 2018). Disease reaction was found to be immune in the case of ‘Pusa Jaikisan *WRR*’. A susceptible disease reaction was found in ‘Pusa Jaikisan’.

### 3.2 Comparative callose deposition

**Figure 2:**
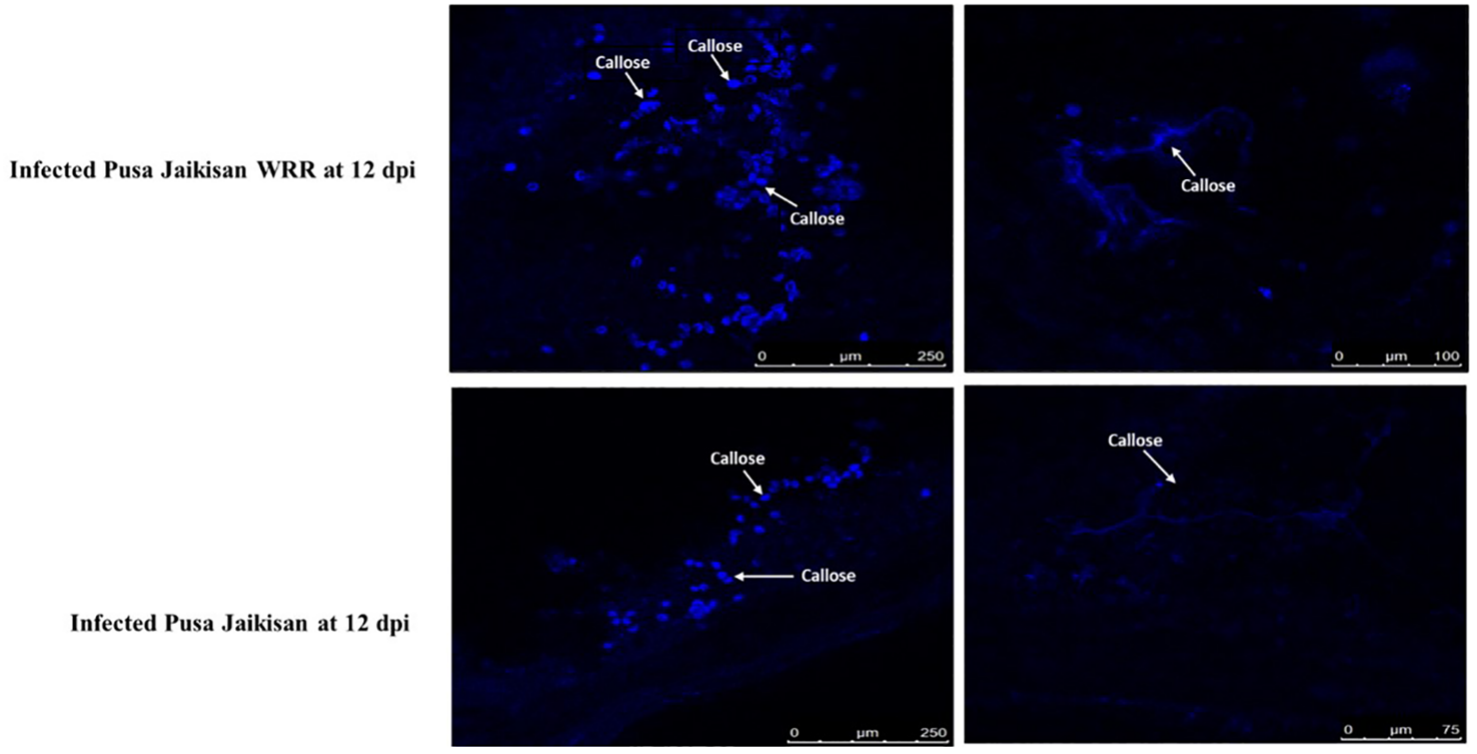
Comparative callose deposition in *B. juncea* cv. Pusa Jaikisan and *B. juncea* cv. Pusa Jaikisan *WRR*. The aniline blue-based histochemical analysis yielded that the callose deposition was higher in ‘Pusa Jaikisan *WRR*’ than in the ‘Pusa Jaikisan’.

Callose deposition is an important determinant of resistance and was histochemically analyzed through aniline blue staining. Based on the confocal microscopic examinations of both the specimens at 12 dpi, i.e., infected ‘Pusa Jaikisan’ and ‘Pusa Jaikisan *WRR*’, we found the callose to be deposited at plasmodesmata, between the plant cell wall and plasma membrane, and on other plant tissues. The callose deposition was observed at different scales i.e., 250 μm and 100 μm for ‘Pusa Jaikisan *WRR*’ and 250 μm and 75 μm in the case of ‘Pusa Jaikisan’. The quantum of callose deposition at the site of pathogen intrusion was found to be way higher in the case of ‘Pusa Jaikisan *WRR*’ as compared to ‘Pusa Jaikisan’ (**Figure 2**). This hinted towards its possible role in the defense against white rust.

### 3.3 Comparative estimation of defense-related biochemical parameters

**Figure 3:**
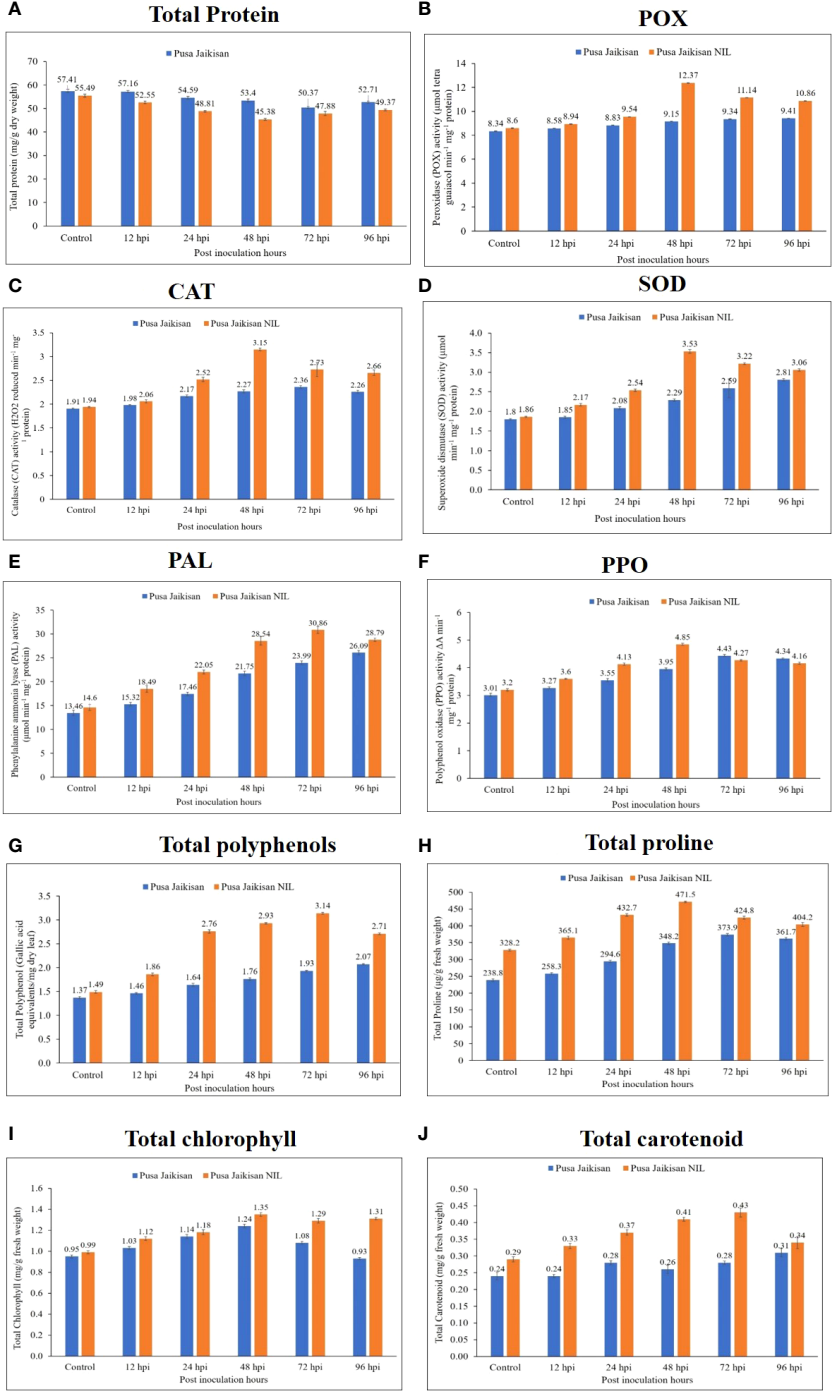
Changes in the biochemical markers in the leaves of ‘Pusa Jaikisan *WRR*’ and ‘Pusa Jaikisan’ after *A. candida* infection at 0, 12, 24, 48, 72 and 96 hpi. Values are mean of three replicates ± SE and indicating significant difference against 0 hpi (p<0.05). The biochemical parameters are **(A)** Total protein; **(B)** POX; **(C)** CAT; **(D)** SOD; **(E)** PAL; **(F)** PPO; **(G)** Total polyphenols; **(H)** Total proline; **(I)** Total chlorophyll; **(J)** Total carotenoids.

Total protein content was higher in ‘Pusa Jaikisan’ than in the ‘Pusa Jaikisan *WRR*’ at all the time points. Across the time intervals, it reduced gradually in ‘Pusa Jaikisan’ but in the case of ‘Pusa Jaikisan *WRR*’, it decreased till 48 hpi and increased after that at both 72 hpi and 96 hpi. However, the protein content of the latter remained lower than the former (**Figure 3A**). Peroxidase was found to be higher in ‘Pusa Jaikisan *WRR*’ as compared to the ‘Pusa Jaikisan’ at all-time intervals. It gradually increased in Pusa Jaikisan from 12 to 96 hpi. But in the case of ‘Pusa Jaikisan *WRR*’, it attained its peak activity at 48 hpi and decreased thereafter (**Figure 3B**). The CAT was observed to be increased in the ‘Pusa Jaikisan *WRR*’ as compared to ‘Pusa Jaikisan’ at all-time intervals. In the case of ‘Pusa Jaikisan *WRR*’, CAT activity was recorded to be highest at 48 hpi and decreased thereafter. It saw a steady rise in the case of ‘Pusa Jaikisan’ (**Figure 3C**). SOD activity was recorded to be higher in ‘Pusa Jaikisan *WRR*’ as compared to ‘Pusa Jaikisan’ at all-time intervals. In the case of ‘Pusa Jaikisan *WRR*’, enzyme activity reached its maximum quantum at 48 hpi and then saw a gradual decline, whereas it kept on increasing at a steady pace in Pusa Jaikisan (**Figure 3D**).

PAL activity was recorded to be higher in the ‘Pusa Jaikisan *WRR*’ as compared to ‘Pusa Jaikisan” at all the time intervals. The enzyme activity was found to be maximum at 72 hpi in the case of ‘Pusa Jaikisan *WRR*’ and decreased at 96 hpi. However, a steady rise in the activity of PAL was seen in ‘Pusa Jaikisan’ (**Figure 3E**). The PPO was recorded to be higher in ‘Pusa Jaikisan *WRR*’ at 12, 24 and 48 hpi, whereas it was slightly higher in ‘Pusa Jaikisan’ at 72 hpi and 96 hpi than the former. The maximum PPO activity in ‘Pusa Jaikisan *WRR*’ was shown at 48 hpi, and a steady decline was seen thereafter. It kept on increasing across all the time points in ‘Pusa Jaikisan’ (**Figure 3F**). In the case of total polyphenol, the activity was found higher in the ‘Pusa Jaikisan *WRR*’ as compared to the ‘Pusa Jaikisan’ at all the time intervals. The activity was found to be maximum at 72 hpi in the case of ‘Pusa Jaikisan *WRR*’ and it decreased at 96 hpi. There was a steady rise in ‘Pusa Jaikisan across all the time points (**Figure 3G**).

The proline content was found to be higher in the ‘Pusa Jaikisan *WRR*’ as compared to the ‘Pusa Jaikisan’. There was a huge difference in the enzyme activity of the 0 hpi sample of both cultivars. In the case of ‘Pusa Jaikisan *WRR*’, the maximum value was attained at 48 hpi followed by a steady decline. It kept on increasing till 72 hpi in ‘Pusa Jaikisan’ and there was a slight decline at 96 hpi (**Figure 3H**). Total chlorophyll content was found to be higher in ‘Pusa Jaikisan *WRR*’ than in ‘Pusa Jaikisan’ at all the time intervals. In the case of Pusa Jaikisan’, the activity increased up to 48 hpi and then decreased from 72 hpi, whereas there was an increment up to 48 hpi followed by a decline at 72 hpi and then again, an increase at 96 hpi in the case of ‘Pusa Jaikisan *WRR*’ (**Figure 3I**). The total carotenoid content was recorded to be higher in ‘Pusa Jaikisan *WRR*’ as compared to the ‘Pusa Jaikisan’ at all the time intervals. In the case of ‘Pusa Jaikisan’, it kept on increasing across the time intervals, whereas in ‘Pusa Jaikisan *WRR*’ it attained the peak value at 72 hpi and decreased thereafter (**Figure 3J**).

**Figure 4:**
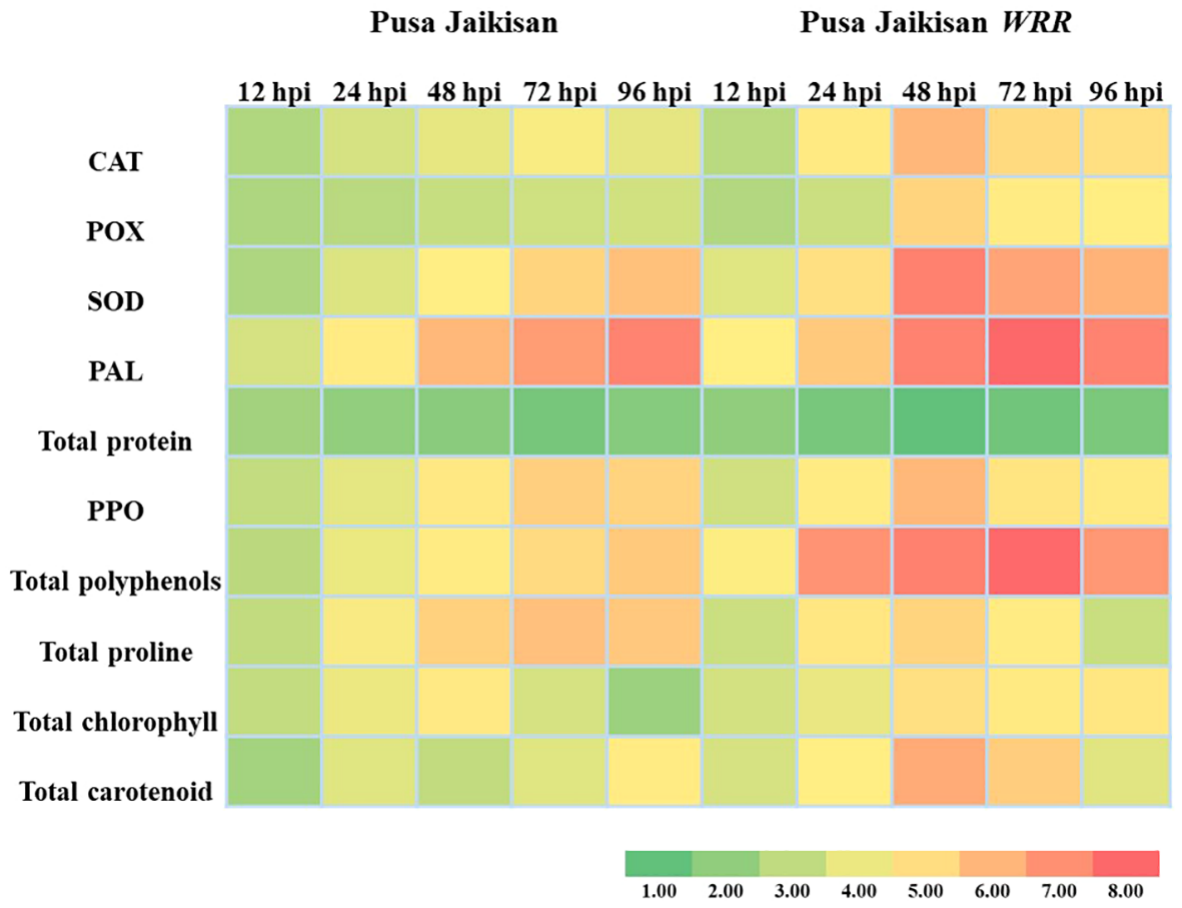
Heatmap analysis of biochemical markers at different time intervals.

The above findings hint towards a positive correlation between resistance and that of antioxidant enzymes (POX, CAT, and SOD), PAL, PPO, polyphenols, proline, chlorophyll, and carotenoids. However, a negative correlation of total protein with disease resistance was observed, which may be due to the breaking down of proteins to produce defense-related enzymes, thereby decreasing protein concentration in plants. Heat map analysis was done for different time intervals against 0 hpi to determine the enzyme with the highest response and best time interval for resistance induction (**Figure 4**). Among all the biochemical markers, SOD, PAL, total polyphenols, and total carotenoids showed a higher quantum of jump as compared to the others. Most of the markers had their highest activity at 48 hpi or 72 hpi. So, these time intervals were the most promising ones for defense response.

### 3.4 Comparative *PR* gene expression

**Figure 5:**
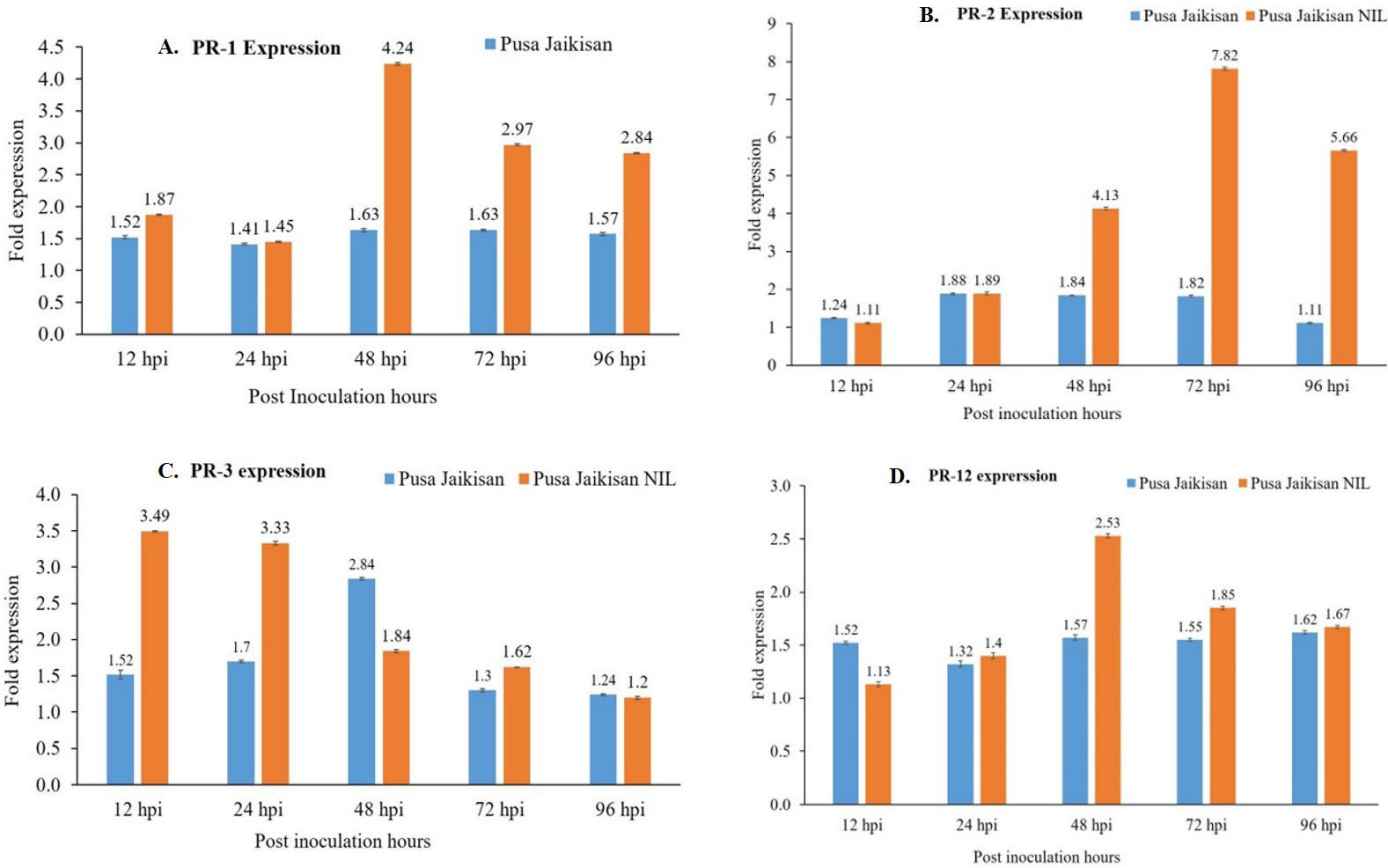
Differential gene expression of *PR1* **(A)**, *PR2* **(B)**, *PR3* **(C)**, and *PR12* **(D)** genes in the leaves of ‘Pusa Jaikisan *WRR*’ and ‘Pusa Jaikisan’ after *A. candida* infection at 0, 12, 24, 48, 72 and 96 hpi. Values are mean of three biological replicates ± SE and indicating significant difference against 0 hpi at p < 0.05. The relative expression levels of each gene were analyzed by delta CT method.

**Figure 6:**
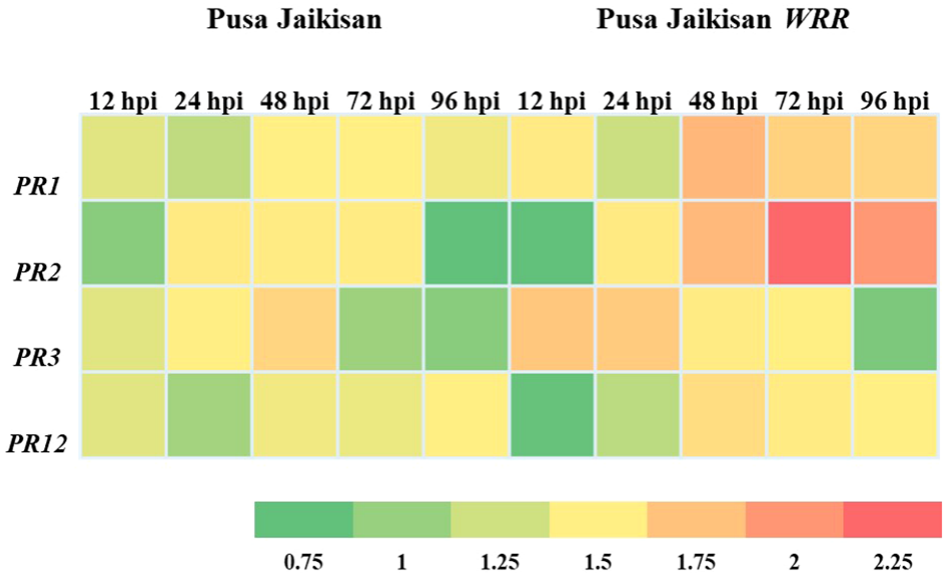
Heat map analysis of PR protein genes at different time intervals.

PR proteins are an important part of the plant’s stress response machinery. *PR1* and *PR*2 are the SA pathway genes which play a vital role in defense against the Biotrophs. The *PR*3 and *PR12* are JA/ET pathway genes, which have a positive role in defense against necrotrophic pathogens. A higher expression of the *PR1* gene was found in the ‘Pusa Jaikisan *WRR*’ as compared to the ‘Pusa Jaikisan’ at all time intervals. In ‘Pusa Jaikisan *WRR*’, the maximum expression (4.24-fold) of *PR1* occurred at 48 hpi. Thereafter, it gradually decreased (2.97-fold at 72 hpi and 2.84-fold at 96 hpi). In ‘Pusa Jaikisan’, the expression was found to be nearly the same at all the time intervals which hovered from a range of 1.41-fold to 1.63-fold (**Figure 5A**). In the case of *PR2* gene expression, ‘Pusa Jaikisan *WRR*’ showed a higher expression at all the time intervals except at 12 hpi as compared to the ‘Pusa Jaikisan’. The highest expression in ‘Pusa Jaikisan *WRR* was seen at 72 hpi (7.82-fold) and decreased at 96 hpi to 5.66-fold. In Pusa Jaikisan, there was not a significant change and the maximum expression occurred at 24 hpi (**Figure 5B**). There was an interesting trend in the JA/ET-marker *PR3* gene expression, where the expression was higher in ‘Pusa Jaikisan *WRR*’ at 12, 24 and 72 hpi, whereas it was reversed at 48 and 96 hpi. In the case of ‘Pusa Jaikisan *WRR*’, maximum expression was seen at 12 hpi (3.49-fold) and 24 hpi (2.84-fold), while for ‘Pusa Jaikisan’ it occurred at 48 hpi (2.84-fold). At 96 hpi, the expression was similar in both cultivars (**Figure 5C**). The expression pattern of *PR12* didn’t show much fluctuation in ‘Pusa Jaikisan’ and hovered around 1.32-fold to 1.62-fold. In ‘Pusa Jaikisan *WRR*’, the highest expression (2.53-fold) occurred at 48 hpi. The resistant cultivar had higher expression at all the time intervals except at 12 hpi. Both the cultivars had at par expression at 96 hpi (**Figure 5D**). The findings of this study hint towards a positive role of *PR1* and *PR2* against *A. candida.* Also, the higher expression of *PR3* in the initial stages and higher expression of *PR12* in ‘Pusa Jaikisan *WRR*’ may be due to their complementary role in resistance to the SA-marker genes. This shows a possible synergistic role of both SA and JA/ET signaling pathways in white rust resistance. Heat map analysis was done for different time intervals against 0 hpi to determine the *PR* gene with the highest response and best time interval for resistance induction (**Figure 6**). Among all the four genes, *PR2* showed the highest relative expression. As with the biochemical markers, gene expression analysis also showed that 48 hpi and 72 hpi are the most active time intervals for defense-response.

## 4 Discussion

The host-pathogen interaction is the most important aspect of any study on plant disease as disease is termed as the manifestation of the interaction between the two entities in a conducive environment. The interaction may be compatible or incompatible based on certain factors out of which host resistance is the foremost. Histological studies are the primary method to establish this binary outcome. The current study emphasizes the role of callose, biochemical markers and PR protein genes in plant defense. In present study deposition of callose in both resistant and susceptible cultivars was observed, but the quantum of deposition was higher in the resistant one. Similar conclusions were made by Kaur et al., 1984, who reported activation of resistance due to the deposition of callose on inner walls. However, there is still a lack of evidence of callose being an important determinant of resistance, as the role may be ascertained due to its easily detectable nature.

The plant-pathogen interaction is characterized by a very well-organized and structured response by the plants to counter the pathogen. Reactive oxygen species (ROS) hold a very important place in *B. juncea*-*A. candida* interaction as the pathogen is an obligate biotroph and the resultant cell death due to the ROS favors the plant. The resistant cultivar ‘Pusa Jaikisan *WRR*’ varied only for a single *R* gene and whatever differential response it showed was due to the transferred gene. Different biochemical parameters were taken viz., total protein, POX, CAT, SOD, PAL, PPO, total polyphenols, total proline, total chlorophyll, and total carotenoids. The total protein and disease resistance in mustard is quite well-studied. Bolwell and coworkers suggested a relationship between the cell wall-invading pathogen and protein content in plants (Bolwell and Wojtaszek, 1997). In our study, we observed the total protein content to be lower in ‘Pusa Jaikisan *WRR*’ as compared to the ‘Pusa Jaikisan’. Similar reports have been made by many researchers. Mishra et al. (2009) evaluated the biochemical response of *Brassica* genotypes against *A. candida* and observed a negative correlation between disease and total proteins. The protein content was maximum in the cotyledonary leaves of susceptible cultivars. Chatterjee et al. (2022) also found fluctuation of significant biochemical parameters such as total proteins, sugars, and phenols, superoxide dismutase, and hydrogen peroxide during the *A. candida* infection in *B. juncea*.

POX catalyzes the oxidation of a variety of phenolic and non-phenolic electron donor substrates with H_2_O_2_ breakdown and is among the first enzymes to show differential response upon induction of biotic stress (Kozłowska et al., 2001). In this study, we observed the POX activity to be higher in the ‘Pusa Jaikisan *WRR*’ than in ‘Pusa Jaikisan’ at all the time intervals. In the resistant cultivar, the maximum activity was seen at 48 hpi followed by a gradual decline. Singh et al. (2011) reported an increment in POX concentration when inoculated with *A. candida*. Faizal canola, a resistant cultivar was found to harbor increased POX activity (Asif et al., 2018). Upadhyay et al. (2023) observed a significant increase in the POX concentration at 48 hpi in all the cultivars upon *A. candida* inoculation. Donskaja, a resistant cultivar showed maximum increase while the susceptible cultivars such as Pusa Bold and Varuna showed a minimal increment. CAT converts the excess H_2_O_2_ produced during developmental and environmental stresses in peroxisome into water and oxygen in all aerobic species. In the present study, CAT activity was found higher in ‘Pusa Jaikisan *WRR*’ than in ‘Pusa Jaikisan’ at all the time intervals. The peak in resistant cultivar was seen at 48 hpi. Similar observations have been made by other researchers. Sapna et al. (2009) found CAT to be higher in the resistant genotype, RH 781 as compared to the susceptible, Varuna upon *A. candida* inoculation. Asif et al. (2018) also noted higher CAT activity in the resistant cultivar, Faisal Canola when challenged with *A. candida*. SOD is an important antioxidant enzyme as it dismutates superoxide radicals into O_2_ and H_2_O_2_. In the present study, the SOD activity was seen to be higher in the resistant cultivar than in susceptible cultivar at all the time intervals. Like the previous antioxidant enzymes, maximum activity in resistant cultivar was seen at 48 hpi. Asif et al. (2018) observed similar results in the resistant cultivars, Faisal canola and Punjab Canola.

PAL catalyzes the deamination process of phenylalanine from the primary metabolism into the crucial secondary phenylpropanoid metabolism in plants. In present study a higher PAL activity was observed in ‘Pusa Jaikisan *WRR*’ than in ‘Pusa Jaikisan’ at all the time intervals. Maximum enzymatic activity was seen at 72 hpi in the ‘Pusa Jaikisan *WRR*’. Singh et al. (1999) had similar findings in which they documented that upon *A. candida* inoculation, *B. juncea* accumulates the PAL. Awasthi (2011) elucidated the positive role of PAL in resistance against white rust as the PAL activity was higher in incompatible reactions as compared to the compatible ones. A negative correlation was also established between PAL concentration and disease severity in the case of Alternaria leaf blight and white rust (Kaur et al., 2020). PPO catalyzes the oxygen-dependent oxidation of phenols to quinone and contributes to the plant defense against plant disease and insect pests. A higher PPO activity was observed in ‘Pusa Jaikisan *WRR*’ than in ‘Pusa Jaikisan’ at 12, 24 and 48 hpi whereas a reverse trend was seen at 72 and 96 hpi. The present findings are in line with Tirmali and Kolte (2013) who concluded that PPO has a positive correlation with the induction of host resistance in otherwise susceptible cultivar, EC-399301 against white rust. Banga et al. (2004) observed a similar trend where they introduced white rust resistance genes into *B. juncea* cv. RL 1359 from *B. napus*, *B. carinata* and *B. tournefortii*. They observed PPO to be positively associated with the white rust-resistant trait.

Phenols are thought to play diverse functions in stressed plants, including the neutralization of ROS, cell wall lignification, and anti-nutritional activity. In the present study total polyphenols content was found to be more in ‘Pusa Jaikisan *WRR*’ than in ‘Pusa Jaikisan’ at all the time intervals. Maximum activity was seen in ‘Pusa Jaikisan WRR’ at 72 hpi. Many researchers have reported similar trends. Yadav et al. (1996) found that total phenols and other phenolic compounds such as polyphenols have a positive role in the Indian mustard against white rust. Phenolic compounds and polyphenols lead to increased deposition of waxes on the leaf surface of *B. juncea* cultivars, and this acted as a structural barrier against the invading *A. candida*. Upadhyay et al. (2023) also showed that the total polyphenols content rises with the disease progression. Donskaja had the maximum polyphenol activity at 24 hpi. Proline is thought to be a potent non-enzymatic antioxidant that can neutralize the detrimental effects of several ROS members. Plants accumulate high amounts of proline in response to stress (Verbruggen and Hermans, 2008). We recorded a higher proline content in ‘Pusa Jaikisan *WRR*’ as compared to the ‘Pusa Jaikisan’ at all the time intervals. There was a huge difference between both cultivars in the proline content at 0 hpi. This hints towards a possible role of proline as an in-built resistance compound. The proline content was highest at 72 hpi and 48 hpi in ‘Pusa Jaikisan’ and ‘Pusa Jaikisan *WRR*’ respectively. Similar reports were also made by Tasleem et al. (2017), where they assessed tolerance to Alternaria blight disease by measuring the activity of oxidative enzymes in a transgenic line (BjV5) of *B. juncea*. Chaurasia et al. (2019) found that in the case of resistant cultivars, A*. brassicae* triggers proteolysis and generates cell-protecting antioxidants as seen in variety, PM-30. This results in higher proline accumulation in this genotype.

In the present study, total chlorophyll content was more in ‘Pusa Jaikisan *WRR*’ as compared to the ‘Pusa Jaikisan’ at all the time intervals. In ‘Pusa Jaikisan’, the activity saw an increment up to 48 hpi and then gradually declined. For ‘Pusa Jaikisan *WRR*’, the activity increased up to 48 hpi, then decreased at 72 hpi and again increased at 96 hpi. These findings are in line with former studies where Gupta et al. (1997) concluded a positive impact of chlorophyll on white rust resistance, and it should be a factor of consideration when screening for white rust resistance genotypes. We recorded and analyzed carotenoid content in both the resistant and susceptible cultivars and found it to be higher in the former at all time intervals. The maximum activity in the resistant cultivar, ‘Pusa Jaikisan *WRR*’ was seen at 72 hpi. Gupta et al. (2012) reported a positive relationship between total carotenoids and the disease intensity in *Alternaria brassicae* infected plants.

The above findings indicate a positive correlation between resistance and that of antioxidant enzymes (POX, CAT, and SOD), PAL, PPO, polyphenols, proline, chlorophyll, and carotenoids. The antioxidant enzymes such as POX, CAT, and SOD and the non-enzymatic ROS scavenging compounds such as polyphenols and proline increase in both resistant and susceptible cultivars, but the quantum of increase is much higher in the former. This may stem from the fact that ROS is an important determinant of cell death, and it occurs much more quickly in resistant cultivars, which ultimately necessitates faster scavenging of ROS to save the plants from excessive cell death. PAL is the first committed enzyme of the cinnamate-related secondary metabolism and is instrumental in resistance. Thus, it was found higher in the resistant cultivars. PPO was found to be increased in the resistant cultivars due to the role it plays in converting the phenols into toxic quinones which further stops the growth of the pathogen. Chlorophyll was reported to increase significantly in both the cultivars. This may be due to the induction of “green islands” by *A*. *candida*, which can fix 5 times more CO_2_ than uninfected plants (Harding et al., 1968). Carotenoids were found in higher concentrations in the resistant cultivar due to the reason that they are potent ROS scavengers. We also reported a negative correlation of total protein with disease resistance. A possible reason may be that for the synthesis of most of the defense-related enzymes, the proteins are broken down thus decreasing their concentration in plants.

PR proteins are an important component of disease resistance along with the hormonal responses that are induced by complex signaling and networking. The PR-protein induction has been reported in many plant-fungi interactions (Grover, 2012). Salicylic acid (SA) and Jasmonic acid (JA)/Ethylene (ET) are two important pillars of hormonal signaling-mediated resistance. Both these are generally thought to be antagonistic. In the case of biotrophs, the SA-mediated resistance is activated while in the case of necrotrophic attack, there is an induction of the JA/ET pathway. The gene-for-gene concept is positively applied to the biotrophs where the interaction between an *Avr* gene and an *R* gene results in resistance. This is further manifested by the activation of SAR and SA-dependent signaling. For this reason, both the concepts were integrated and *PR1* and *PR2* were taken as SA-marker genes and *PR3* and *PR12* were taken as JA/ET-marker genes to check whether both pathways are synergistic or antagonistic and which set of PR genes play a positive role in white rust resistance against *B. juncea*.

We found that the resistant cultivar ‘Pusa Jaikisan *WRR*’ had a higher expression of the *PR1* gene at all the time intervals as compared to the susceptible one. In the resistant cultivar, the maximum expression occurred at 48 hpi. Thereafter, it gradually declined. In the case of ‘Pusa Jaikisan’, the expression was found to be similar at all the time intervals. Similar findings were observed by other workers such as Niderman et al. (1995) reported the *PR1* protein isolated from tobacco and other solanaceous plants effectively reduced spore germination and pathogen growth in the plants. Overexpression of numerous *PR1* genes in various plant species increased resistance to oomycetes (Broekaert et al., 2000), but the effect on other pathogens taxa is unknown. *PR1* has recently been demonstrated to bind sterols, indicating a protective mode of action based on the limitation of this key nutrient for oomycetes (Gamir et al., 2017). In the case of *PR2* gene expression, the ‘Pusa Jaikisan *WRR*’ showed a higher relative expression than in ‘Pusa Jaikisan’ at all the time intervals except at 12 hpi. Maximum expression in ‘Pusa Jaikisan *WRR*’ occurred at 72 hpi. There was not much significant rise in the ‘Pusa Jaikisan’ and the expression was relatively the same throughout. Several incidences of improved resistance to oomycetes in plants overexpressing *PR2* have been documented since oomycetes have β-1,3-Glucanase in their cell walls (Broekaert et al., 2000).

Chitinase (*PR3*) catalyzes the hydrolytic cleavage of chitin (the planet’s second most prevalent biopolymer after cellulose) and is a significant antifungal enzyme We saw a very interesting trend in *PR3* gene expression where it was more in ‘Pusa Jaikisan *WRR*’ at 12,24 and 72 hpi, while at 48 and 96 hpi it was more in the susceptible cultivar, ‘Pusa Jaikisan’. Maximum expression in ‘Pusa Jaikisan *WRR*’ occurred in the initial stages at 12 and 24 hpi. For the ‘Pusa Jaikisan *WRR*’, the peak was observed at 48 hpi. At 96 hpi both had a very similar expression. *PR12* (also known as ‘plant defensin’) are the most important PR proteins for necrotrophic pathogen resistance. The expression pattern of *PR12* in the current study didn’t show much fluctuation in the ‘Pusa Jaikisan’. In ‘Pusa Jaikisan *WRR*’, the peak expression was seen at 48 hpi. The ‘Pusa Jaikisan *WRR*’ had higher expression at all time points except at 12 hpi. At 96 hpi, just like the *PR3*, *PR12* also had similar expression in both the cultivars. The role of *PR3* and *PR12* in disease resistance was elucidated by Yadav et al. (2020) as they conducted a gene expression analysis that revealed upregulation of *PR3* and *PR12* genes only in *C. sativa* and *S. alba* as compared to *B. juncea*, implying their role in Alternaria resistance. This may hint towards the involvement of *PR3* in general resistance mechanism against both biotrophs and necrotrophs.

The above findings indicate a positive role of *PR1* and *PR2* genes in the white rust resistance of *B. juncea*. Also, the higher expression of *PR3* in the initial stages and higher expression of *PR12* in the resistant cultivar may be due to the positive role of JA/ET signaling that acts complementary to the SA pathway. Due to the tendency of SA and JA/ET signaling to be antagonistic to one another, JA/ET signaling is anticipated to negatively impact resistance to these pathogens. These findings are in line with the observation of Glazebrook (2005), who reported that the SA signaling is crucial for resistance against biotrophs such as *Peronospora parasitica*, *Erysiphe* species and *Pseudomonas syringae*. However, JA/ET signaling may also be instrumental in resistance if it is active, particularly in the case of *P. parasitica* and *Erysiphe* species. The biotrophs are generally known to be stopped by SA signaling and JA/ET acts against necrotrophs and insects. These two pathways are thought to be antagonistic but there is a lot of evidence that the JA/ET pathway is potentiating the SA pathway in resistance against the biotrophs as well. Incompatible reaction in *Plasmopara viticola*, a biotrophic oomycete was mediated through the JA pathway (Guerreiro et al., 2016). This may be the case with *A. candida*- *B. juncea* interaction, where both pathways possibly play synergistic roles.

## 5 Conclusion

*B. juncea* is one of the most important edible oilseed crops and *A. candida* is a serious impediment in realizing its production potential. Given the pathogen’s status of being an obligate biotroph, the *R* gene based resistance is easily overcome by more virulent races. So, to develop durable resistant varieties, we need to explore the factors behind resistance. In the present study, we studied the morphological, histochemical, biochemical, and molecular parameters and their role in white rust resistance in resistant (Pusa Jaikisan WRR) and susceptible (Pusa Jaikisan) cultivars of *B. juncea*. Morphological studies confirmed the resistant nature of ‘Pusa Jaikisan *WRR*’ as there was no spot on either the adaxial or abaxial surface. Histochemical studies hinted towards a positive role of callose deposition in resistance as it was deposited in a higher quantum in ‘Pusa Jaikisan *WRR*’. Biochemical studies established a positive correlation between resistance and POX, CAT, SOD, PAL, PPO, polyphenols, proline, chlorophyll, and carotenoids, while there was a negative correlation between total protein and white rust resistance. In addition to these, the gene expression analysis revealed that the SA-marker genes played a crucial role in resistance, while the JA-responsive genes may also have a positive role in the white rust defense mechanism. The comparative histochemical, biochemical, and molecular studies showed that the ‘Pusa Jaikisan *WRR*’ activated defense response by early detection of the pathogen. Results obtained in this study will pave the way for developing strategies to induce resistance against biotrophic pathogens in susceptible cultivars by developing transgenic plants over-expressing the *PR1* and *PR2* genes. The role of *PR3* and *PR12* should also be established in the case of other biotrophic pathogens of *B. juncea* such as *Erysiphe polygoni* and *Hyaloperonospora parasitica*. If the role of these JA-marker genes is positive in these cases as well, then these genes can be utilized by over-expression for a broad-spectrum resistance against both biotrophic and necrotrophic pathogen complexes of *B. juncea*.

## Data availability statement

The authors confirm that the data supporting the findings of this study are available within the article. The data is part of thesis submitted in MS Swaminathan Library (T-11213). Raw data supporting the findings of this study are available from the corresponding author upon resonable request.

## Author contributions

PR: Conceptualization, Data curation, Formal analysis, Funding acquisition, Investigation, Resources, Software, Writing – original draft, Writing – review & editing. LP: Conceptualization, Data curation, Formal analysis, Supervision, Writing – review & editing. SM: Methodology, Supervision, Writing – review & editing. PY: Methodology, Supervision, Validation, Writing – review & editing. AS: Data curation, Formal analysis, Methodology, Resources, Supervision, Writing – review & editing. DM: Data curation, Methodology, Supervision, Validation, Writing – review & editing.
